# Analysis of plasma metabolic biomarkers in the development of 4-nitroquinoline-1-oxide-induced oral carcinogenesis in rats

**DOI:** 10.3892/ol.2014.2619

**Published:** 2014-10-15

**Authors:** XIANGLI KONG, XIAOQIN YANG, JINGLIN ZHOU, SIXIU CHEN, XIAOYU LI, FAN JIAN, PENGCHI DENG, WEI LI

**Affiliations:** 1 State Key Laboratory of Oral Diseases, West China Hospital of Stomatology, Sichuan University, Chengdu, Sichuan 610041, P.R. China; 2 Department of Oral and Maxillofacial Surgery, Guangdong Provincial Stomatological Hospital, Southern Medical University, Guangzhou, Guangdong 510515, P.R. China; 3 Analytical and Testing Center, Sichuan University, Chengdu, Sichuan 610041, P.R. China

**Keywords:** metabolomics, 4-nitroquinoline-1-oxide, oral squamous cell carcinoma, oral leukoplakia

## Abstract

The aim of the present study was to identify time-dependent changes in the expression of metabolic biomarkers during the various stages of oral carcinogenesis to provide an insight into the sequential mechanism of oral cancer development. An ^1^H nuclear magnetic resonance (NMR)-based metabolomics approach was used to analyze the blood plasma samples of Sprague-Dawley rats exhibiting various oral lesions induced by the administration of 4-nitroquinoline-1-oxide (4NQO) in drinking water. The ^1^H NMR spectra were processed by principal component analysis (PCA) and partial least-squares discriminant analysis (PLS-DA) to determine the metabolic differences between the three developmental stages of oral mucosa cancer (health, oral leukoplakia [OLK] and oral squamous cell carcinoma [OSCC]). The variable importance in projection (VIP) score derived from the PLS-DA model was used to screen for important metabolites, whose significance was further verified through analysis of variance (ANOVA). Data from the present study indicated that 4NQO-induced rat oral carcinogenesis produced oral pre-neoplastic and neoplastic lesions and provided an effective model for analyzing sequential changes in the ^1^H NMR spectra of rat blood plasma. The ^1^H NMR-based metabolomics approach clearly differentiates between healthy, OLK and OSSC rats in the PCA and PLS-DA models. Furthermore, lactic acid, choline, glucose, proline, valine, isoleucine, aspartic acid and 2-hydroxybutyric acid demonstrated VIP>1 in the PLS-D model and P<0.05 with ANOVA. It was also identified that increases in lactic acid, choline and glucose, and decreases in proline, valine, isoleucine, aspartic acid and 2-hydroxybutyric acid may be relative to the characteristic mechanisms of oral carcinogenesis. Therefore, these plasma metabolites may serve as metabolic biomarkers in oral carcinogenesis and assist in the early diagnosis and preventive treatment of oral cancer.

## Introduction

Advanced-stage oral cancer is associated with high incidences of mortality, morbidity and disfigurement worldwide. Therefore, it is important that oral cancer is detected early for improved prevention, prognosis and treatment (1). Recently, a number of novel techniques have been developed that aid with the early detection of oral cancer (2), for example metabolomics assesses and validates metabolite concentrations within cells, tissues and biofluid. In addition, it has been applied to investigate the underlying mechanisms and, thus, the early detection of oral cancer (3–8).

Metabolomic analyses of the biofluid of oral cancer patients (such as urine, serum and saliva) have been performed using differential measurement techniques, including nuclear magnetic resonance (NMR), high performance liquid chromatography-mass spectrometry (HPLC-MS) and gas chromatography (GC)-MS. Additionally, data were processed using multivariate statistical analyses, such as principal component analysis (PCA) and partial least-squares discriminant analysis (PLS-DA). Previous studies, including our previous study (3–7) have analyzed human biofluid to demonstrate the applicability of metabolomics in the diagnosis and prognosis of oral cancer. However, metabolomics is sensitive to a number of variables, including host susceptibility and resistance, diet, lifestyle and geographical location (9). Difficulty identifying the metabolic markers of oral cancer and metabolic differences between the stages of oral carcinogenesis (healthy, oral leukoplakia [OLK] and oral squamous cell carcinoma [OSCC]) may be due to variations in the above-mentioned human variables.

A 4-nitroquinoline-1-oxide (4NQO) rat model of oral carcinogenesis exhibits all stages of oral carcinogenesis, and has been reported as histologically and molecularly similar to human oral carcinogenesis (10,11). In addition, a rat model allows conditions to be maintained with limited variation, providing reproducible isolation of all stages of carcinogenesis. Therefore, 4NQO-induced rats are an effective model for the investigation of various metabolites in the neoplastic progression of oral cancer.

The aim of the present study was to identify potential biomarkers for the diagnosis and classification of OSCC and precancerous lesions by identifying important plasma metabolites, as well as analyzing their concentration trends in the principal stages of oral carcinogenesis via 4NQO-induced oral carcinogenesis in rats.

## Materials and methods

### Animal treatment and sampling

Forty male, specific pathogen-free Sprague-Dawley rats (age, eight weeks; weight, ~250 g) were obtained from the Experimental Animal Center of Sichuan University (Chengdu, China). The rats were maintained under controlled conditions at 24±2°C on a 12-h light-dark cycle and with free access to water and a commercial diet. (rat and mouse feed; Dasuo Bio-Technology Ltd., Chengdu, China) Following one week of acclimatization, the rats were randomly divided into two groups: A 4NQO-treated group, n=30 (treatment, 50 parts per million [ppm] 4NQO solution); and a healthy control group, n=10 (treatment, water). Following 16 and 24 weeks of 4NQO and water treatment, the rats were sacrificed by intraperitoneal injection of 3 ml/kg sodium pentobarbital (Sigma-Aldrich, St. Louis, MO, USA) and autopsied. The rats were divided into three groups as demonstrated in Table I. A total of 30 plasma samples (1 ml of plasma for each sample) were obtained and stored at −80°C prior to ^1^H NMR spectroscopic analysis. Tongue samples were collected for histopathological examination.

All experimental protocols were approved by the Animal Experimental Ethics Committee of Sichuan University (Chengdu, China) and conformed to procedures described in the Guiding Principles for the Use of Laboratory Animals (12).

### Histopathological evaluation

The whole tongue was excised, flattened on a transparency plate and fixed in 4% phosphate-buffered saline-buffered formalin. The formalin-fixed tongues were then cut into four sections, processed and embedded in paraffin. The 5-μm thick sections were stained with hematoxylin and eosin for histopathological analysis. OLK and OSCC were diagnosed according to the World Health Organization classification of tumors (2005) (13) and Warnakulasuriya *et al* (14).

### Sample preparation and NMR spectroscopy

Samples were prepared by mixing plasma (250 μl ) with 99.9% deuterium oxide (250 μl) and leaving to stand for 10 min to obtain a deuterium lock signal for NMR spectrometry. The mixtures (500 μl) were then transferred into 5-mm NMR capillary tubes. ^1^H NMR spectra were measured at 600.13 MHz on a Bruker Avance™ II 600 spectrometer (Bruker Biospin, Rheinstetten, Germany) using a 5-mm PATXI probe at a temperature of 300 K. The spectra were acquired using a one dimensional (1D) Carr-Purcell-Meiboom-Gill (CPMG) pulse sequence (CPMGpr), which attenuated the broad protein signal in the plasma. This sequence is a modification of the CPMG pulse sequence that suppresses the residual water signal (15). For each sample, the 1D ^1^H NMR spectrum was collected with 64 K data points, 64 scans and 15-ppm spectral width. Furthermore, the acquisition parameters included a 5-μsec relaxation delay, eight dummy scans, 400-μsec fixed echo time to allow the elimination of J-modulated spin-echo, and 400 CPMG loops for T2 filter.

Data were processed using MestReNova software 6.0.4 (Mestrelab Research, S.L, Santiago de Compostela, Spain). The NMR spectra acquired were manually phased, corrected for baseline distortion using the Whittaker smooth algorithm (16), and referenced to the lactic acid doublet at 1.30 and 4.11 ppm. The spectral regions of residual water were excluded from analysis (4.3–6.5 ppm). The spectral region of δ9.00-0.50 was segmented into 314 bins (width, 0.02 ppm). The resulting 314 integrals were normalized to the sum of the spectral intensity to compensate for differences in the concentrations of the samples. Metabolites were identified by comparing their ^1^H chemical shifts and coupling patterns with the corresponding values from the Human Metabolome Data Bank (http://www.hmdb.ca).

### Multivariate statistical analysis

Following data normalization, the data set was input into SIMCA-P 11.5 software (Umetrics, Umeå, Sweden), pareto-scaled in a column-wise manner and processed using the unsupervised method of PCA. This provided an analysis of the overall distribution of the data set and was used to identify and eliminate abnormal samples from the three groups. The supervised method of PLS-DA was also used to maximize variation between the three groups and to identify the metabolites important in oral carcinogenesis.

The results are presented as principal component score plots, with each point in the plot representing an individual sample. The quality of the PCA and PLS-DA models is depicted by the cross-validation parameters, R2 and Q2, representing the explained variance and the predictive capability of the model, respectively. R2X and R2Y represent the fraction of variance of the X and Y matrix, respectively, and Q2Y represents the predictive accuracy of the model, with cumulative (cum) values of R2X, R2Y and Q2Y equating to ~1 indicating an effective model. Furthermore, permutation tests (200 cycles) were conducted to assess the robustness of the PLS-DA model when using a small sample size (17).

Discriminant plasma metabolites from the OSCC, OLK and control groups were defined by a statistically significant threshold of variable importance in the projection (VIP), which was derived from the PLS-DA model. VIP>1.0 was considered sufficient for group discrimination (18).

### Univariate statistical analysis

Univariate statistical analysis was used to verify the significance of the metabolites that were screened using multivariate statistical analysis. Online software (www.metaboanalyst.ca) (19) was applied to perform an analysis of variance (ANOVA) and P<0.05 was considered to indicate a statistically significant difference. Thus, the variables selected were those with VIP>1 and P<0.05 according to PLS-DA and ANOVA, respectively.

## Results

### 4NQO-induced oral carcinogenesis

**Figure 1:**
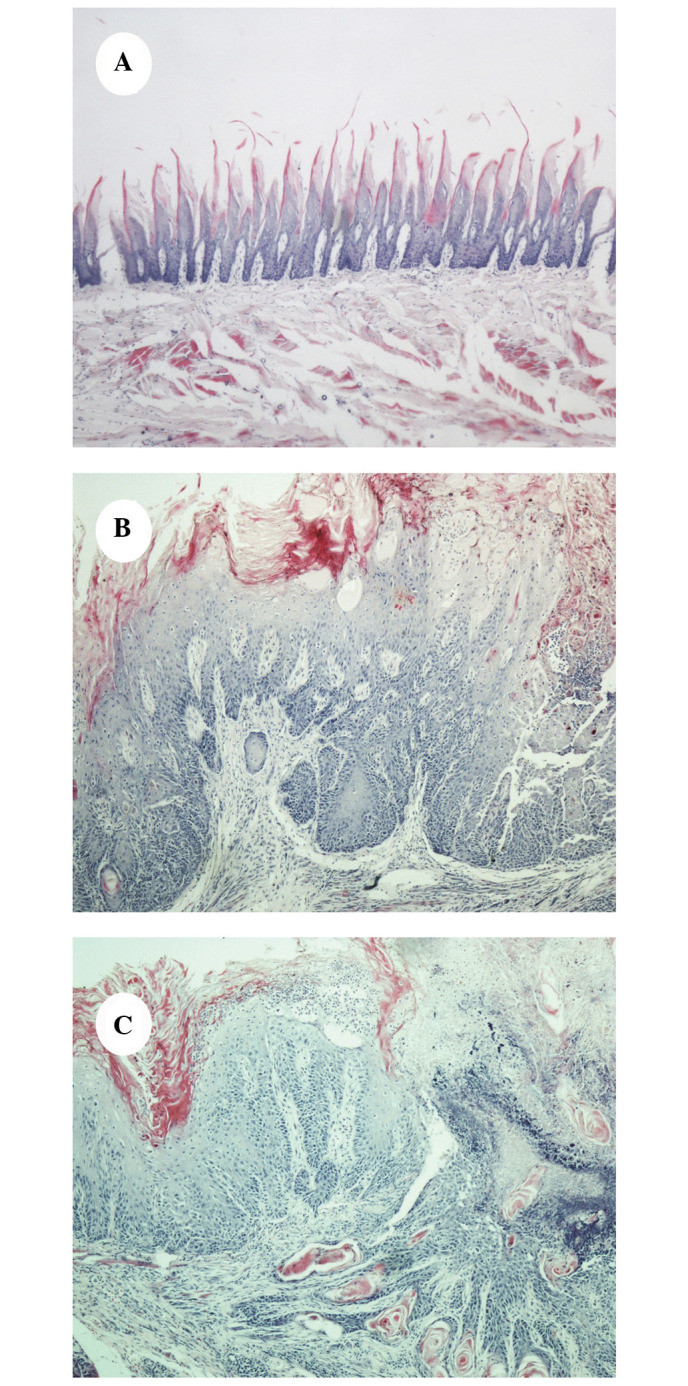
Histopathological images of 4-nitroquinoline-1-oxide-induced oral lesions on rat tongues in the (A) control, (B) oral leukoplakia and (C) oral squamous cell carcinoma group (hematoxylin and eosin staining; magnification, ×100).

Prolonged application of the carcinogenic agent (4NQO) caused the tongue mucosa of the 4NQO-treated rats to present with varying degrees of dysplasia. All rats in the healthy control group appeared histologically normal. The majority of the 4NQO-treated rat tongue mucosas exhibited hyperemia and ulceration at weeks 10–12; a visibly rough, granular mucosal surface with varying degrees of erythema, as well as occasional white plaque-like lesions were observed at weeks 14–16 and OSCC presented at weeks 22–24. The typical histopathology of the 4NQO-induced oral lesions of the control, OLK and OSCC groups are demonstrated in Fig. 1A–C, respectively.

### ^1^H NMR spectra of the rat plasma samples

**Figure 2:**
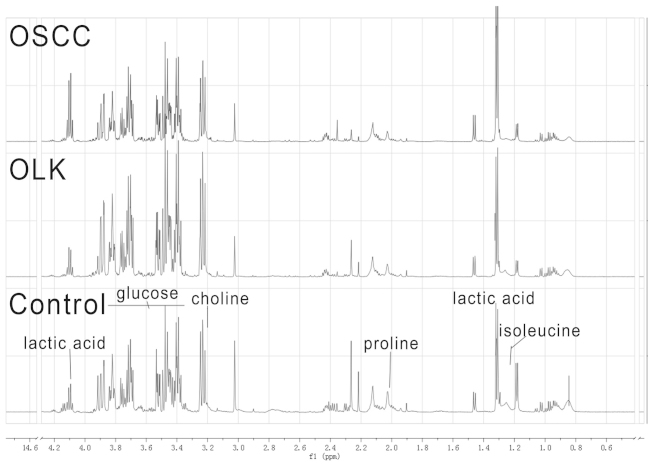
Representative ^1^H nuclear magnetic resonance spectra of rat plasma at various stages of oral carcinogenesis. OSCC, oral squamous cell carcinoma; OLK, oral leukoplakia.

Fig. 2 depicts the representative ^1^H NMR spectra, which demonstrates the common signals from the control, OLK and OSCC group samples. The spectra are dominated by carbohydrate resonances, in particular, the two anomeric forms of glucose (3.0–5.5 ppm) and various intermediate metabolites of the glycolytic pathway, such as lactic acid (1.31 and 4.10 ppm). Other compounds, in particular the amino acids, valine (0.9–1.1 ppm) and proline (2.02 ppm), exhibit large methyl signals in the spectrum.

### Multivariate statistical analysis

**Figure 3:**
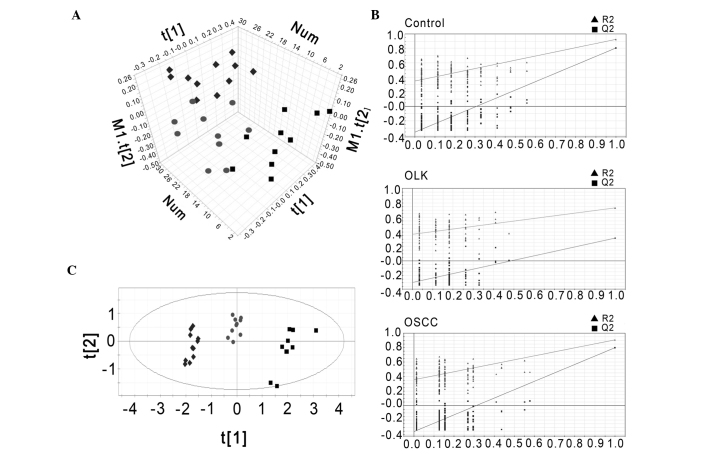
(A) Principal component analysis score plot of ^1^H NMR spectra of rat plasma. (B) Validation plots of the PLS-DA model for rat plasma using a permutation test that was randomly permuted 200 times with the first principal component (▲, R2; ■, Q2). (C) PLS-DA scatter plot derived from ^1^H NMR spectra of rat plasma samples (groups: Control, ■; ●, OLK; and ◆, OSSC). OLK, oral leukoplakia; OSCC, oral squamous cell carcinoma; PLS-DA partial least-squares discriminant analysis; NMR, nuclear magnetic resonance; R2, explained variance; Q2, predictive capability of the model.

The data set from the ^1^H NMR spectra was processed using the unsupervised method of PCA using SIMCA-P 11.5 software (Umetrics) to generate an unbiased overview of the major metabolic differences between the control, OLK and OSCC groups. As indicated in Fig. 3A, three groups exhibited a trend of intergroup separation on the score plot (R2X[cum]=70.6%; Q2[cum]=56.3%).

To obtain a more reliable statistical analysis and specific loadings, a PLS-DA model was used to discriminate samples from the control, OLK and OSCC groups. A cluster of 200 permutated models from the first component were visualized using validation plots (Fig. 3B). In the permutation test, all permutated R2 and Q2 values to the left were lower than the original point to the right, and lower than the original values, which is an indication of the validity of the original models. According to VIP, the most significant discriminatory metabolites between the three groups were lactic acid (VIP, 12.15), choline (VIP, 3.73), and glucose (VIP, 2.22), and proline (VIP, 3.55), valine (VIP, 3.27), isoleucine (VIP, 2.89), aspartic acid (VIP, 2.12), glutamine (VIP, 2.02) and 2-hydroxybutyric acid (VIP, 1.78). The plasma concentrentions of lactic acid, choline, and glucose were increased, while those of proline, valine, isoleucine, aspartic acid, glutamine and 2-hydroxybutyric acid were decreased. The score plot is presented in Fig. 3C, and demonstrates three distinct clusters (the OSCC, OLK and control groups; R2X=0.705; R2Y=0.787; Q2[cum]=0.641).

### ANOVA of discriminatory metabolites in the plasma

**Figure 4:**
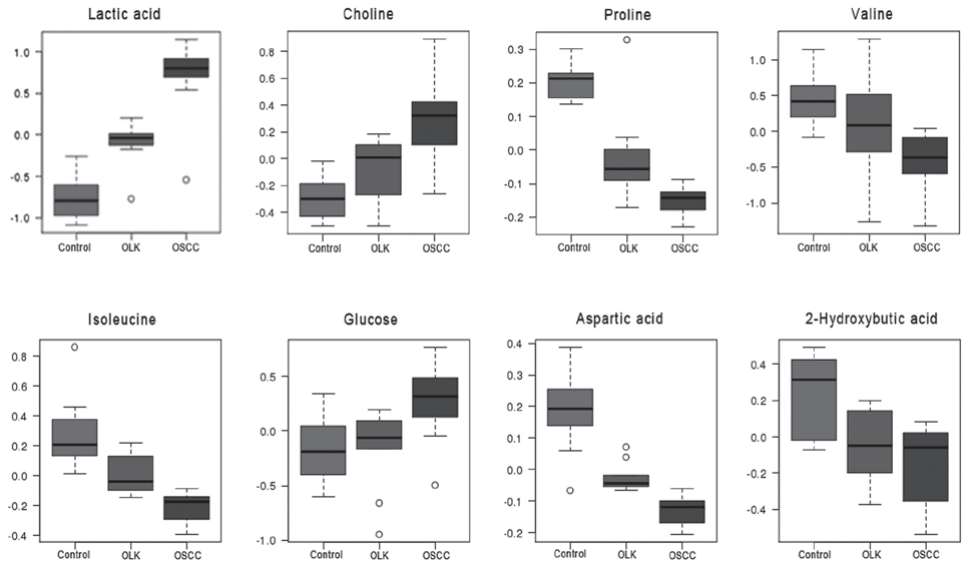
Box plots of the concentration variations of eight discriminant metabolites in rat plasma at various stages of oral carcinogenesis derived from analysis of variance (P<0.05). Empty circles present anomalous results.

ANOVA was applied to to further verify the significance of the discriminatory metabolites screened in PLS-DA (VIP>1). The ANOVA results were generally consistent with those that were derived from the PLS-DA model, with the exception of glutamine, which demonstrated no significant difference between the three groups. The discriminant metabolites, lactic acid, choline, and glucose increased, whereas proline, valine, isoleucine, aspartic acid and 2-hydroxybutyric acid decreased in the plasma at various stages of oral carcinogenesis (Fig. 4).

## Discussion

In the present study, PCA and PLS-DA were used to analyze the ^1^H NMR-spectra of plasma using a 4NQO-induced oral carcinogenesis rat model and to identify metabolic biomarkers in the development of oral cancer. The data from the present study indicates that ^1^H NMR-based metabolomic analyses of plasma distinguish between OSCC, OLK and healthy rats. Furthermore, using the rat model, significant increases in lactic acid, choline, and glucose (P<0.05), and significant decreases in proline, valine, isoleucine, aspartic acid and 2-hydroxybutyric acid were observed in the plasma during 4NQO-induced oral carcinogenesis, indicating that the changes of these eight metabolites may be related to each other and viewed as a concentration profile which may be important in the development of oral cancer.

The development of oral cancer is a multistep process, which includes a sequence of changes from hyperplasia through to dysplasia (leukoplakia) and finally to carcinoma (20). The majority of recent metabolic studies of oral cancer have focused on patients’ biofluid, such as urine, serum and saliva. For example, Tiziani *et al* (4) used PCA and PLS-DA to investigate metabolic data derived from serum NMR spectroscopy of OSCC patients and healthy subjects. The serum ^1^H NMR spectra of the OSCC patients were clearly distinguishable from those of the healthy controls, and 23 differential metabolites were determined. Our previous study applied ^1^H NMR-based metabonomic methods to investigate the differences in plasma metabolite concentration between OSCC, OLK and healthy control groups. Furthermore, various discriminatory metabolites were identified and PLS-DA analysis was applied to determine an effective model for the detection of ^1^H NMR data that differentiates OSCC patients from OLK or control patients (5). Salivary and urinary metabonomic analyses have also been conducted using HPLC-MS or GC-MS and have demonstrated that various saliva- or urine-derived metabolites may serve as diagnostic tools for the early detection of oral cancer (6,8). However, studies regarding discriminatory metabolites have produced conflicting results, possibly due to variations within individuals analyzed in the studies, which may hinder the analysis of metabolite biomarker concentration during oral carcinogenesis. In the present study, animal models were raised in controlled conditions, avoiding the possible variations that are observed in the human studies and aiding with the analysis of metabolic biomarkers in oral carcinogenesis.

Wei *et al* (8) reported a metabolomic study using a classical model of 7,12-dimethylbenz(a)anthracene (DMBA)-induced oral carcinogenesis in hamsters to delineate characteristic metabolic transformation during carcinogenesis using GC time-of-flight MS. The animal model of oral carcinogenesis provided a reproducible and continuous model for reducing the impact of human variables. Although the study revealed various differential metabolites, they were only identified between two groups (healthy vs. OLK subjects; healthy vs. OSCC subjects; or OLK vs. OSCC subjects) rather than between three groups (healthy vs. OLK vs. OSCC subjects), which may not represent the continuous change of discriminatory metabolites during oral carcinogenesis.

In the present study, a 4NQO-induced oral carcinogenesis model was used to identify the metabolic biomarkers of oral cancer development. This method is simpler and safer than surgery and avoids the mechanical stimulation resulting from the use of a needle. The 4NQO-induced model of oral carcinogenesis has been applied in numerous studies, and is a classic and effective model, which is histologically and molecularly similar to human oral carcinogenesis.

The present study identified a gradually increasing plasma concentration of lactic acid, choline and glucose, and a stepwise reduction in the plasma concentration of proline, valine, isoleucine, aspartic acid and 2-hydroxybutyric acid in healthy, OLK and OSCC groups, indicating a time-dependent progression of metabolic changes that are associated with oral carcinogenesis.

Tiziani *et al* (4) identified that lactic acid concentration decreased in human patients exhibiting OSCC; however, a different study identified that lactic acid concentration increased in hamsters exhibiting dysplasia and OSCC (8), which is consistent with the observation of 4NQO-induced oral carcinogenesis in rats in the present study. An elevated lactic acid concentration may be associated with the Warburg effect (21), tumor hypoxia (22) and the low pH of the tumor microenvironment (23). Furthermore, consistent with Tiziani *et al* (4), the present study observed relatively high concentrations of glucose, possibly due to an increase in the demand of carbon skeletons and energy required to sustain metabolic activities (24). Therefore, elevated glucose uptake and enhanced glycolytic pathway activity resulted in increased glucose and lactic acid concentrations during oral carcinogenesis. However, in contrast to Tiziani *et al* (4), the present report identified a decrease in 2-hydroxybutyric acid concentration during the development of oral cancer. This may be due to the decrease in aerobic oxidation resulting in a reduction in the NADH_2_/NAD ratio (25).

In the present study, amino acids, including valine, isoleucine, aspartic acid and proline were observed to gradually decline during the development of oral cancer. Valine and isoleucine are branched-chain amino acids, which are supplemented by food uptake and used preferentially during tumor development (26). The present study identified a decrease in valine and isoleucine concentration, possibly due to difficulty in food uptake as a result of tongue damage and increased metabolic utilization during oral carcinogenesis. A reduction in aspartic acid and proline concentration was also observed that may have been due to their role in the tricarboxylic acid cycle, which is upregulated in response to the increased demand for glucose and protein synthesis during cancer cell proliferation.

Various studies have demonstrated that increases in choline-associated metabolites are associated with genetic alterations typically detected in cancer cells (27). Choline-associated metabolites are increasingly being used as an adjunct in the diagnosis of primary malignant tumors of the brain (28), prostate (29) and breast (30). Consistent with Tiziani *et al* (4), the present study identified a stepwise elevation in the plasma choline concentration of OSCC patients during 4NQO-induced oral carcinogenesis. Choline, as well as lactic acid, glucose, 2-hydroxybutyric acid, valine, isoleucine, aspartic acid, and proline exhibited a distinct differentiation pattern in healthy, OLK and OSCC groups. This differentiation pattern indicates that these metabolites may be used in combination as metabolic biomarkers for discriminating between OSCC, OLK and healthy subjects.

In conclusion, the 4NQO-induced rat model of oral carcinogenesis is an effective model for analyzing metabolic biomarkers in the development of oral cancer using ^1^H NMR spectroscopy, PCA, PLS-DA and ANOVA. A concentration profile of the healthy, OLK, and OSCC groups was formed by measuring the change in lactic acid, glucose, 2-hydroxybutyric acid, valine, isoleucine, aspartic acid, proline and choline concentrations. This concentration profile may be a valuable tool for discriminating between OSCC, OLK, and healthy subjects or OLK and healthy subjects, as well as for predicting the occurrence and development of oral cancer. However, further research should be combined with various analytical techniques, such as LC-MS, GS-MS, and different types of biofluid, such as urine and saliva, to obtain systematic metabolic differences at all stages of oral cancer. Investigating the association between metabolites and the genes or proteins involved in the relative metabolic pathways of oral cancer may assist in providing a metabolic mechanism for oral carcinogenesis.

## Figures and Tables

**Table I:** Rat groups and treatment strategies.

Group	Treatment	Time, weeks	Rats, n
Control (n=10)	Water	16	5
		24	5
OLK	50 ppm 4NQO solution	16	10
OSCC	50 ppm 4NQO solution	24	10

OLK, oral leukoplakia; ppm, parts per million; 4NQO, 4-nitroquinoline-1-oxide; OSCC, oral squamous cell carcinoma; OLK, oral leukoplakia; ppm, 4NQO, 4-nitroquinoline-1-oxide; OSCC, oral squamous cell carcinoma.
